# Natural history of model organisms: The secret (group) life of *Drosophila melanogaster* larvae and why it matters to developmental ecology

**DOI:** 10.1002/ece3.7003

**Published:** 2020-11-10

**Authors:** Juliano Morimoto, Zuzanna Pietras

**Affiliations:** ^1^ School of Biological Sciences University of Aberdeen Aberdeen UK; ^2^ Department of Physics, Chemistry and Biology (IFM) Linköping University Linköping Sweden

**Keywords:** diet breadth, ecological specialization, Niche, polyphagy

## Abstract

1. Model organisms such as *Drosophila melanogaster* have been key tools for advancing our fundamental and applied knowledge in biological and biomedical sciences. However, model organisms have become intertwined with the idea of controlled and stable laboratory environments, and their natural history has been overlooked.
2. In holometabolous insects, lack of natural history information on larval ecology has precluded major advances in the field of developmental ecology, especially in terms of manipulations of population density early in life (i.e., larval density). This is because of relativistic and to some extent, arbitrary methodologies employed to manipulate larval densities in laboratory studies. As a result, these methodologies render comparisons between species impossible, precluding our understanding of macroevolutionary responses to population densities during development that can be derived from comparative studies.
3. We recently proposed a new conceptual framework to address this issue, and here, we provide the first natural history investigation of *Drosophila melanogaster* larval density under such framework. First, we characterized the distribution of larval densities in a wild population of *D. melanogaster* using rotting apples as breeding substrate in a suburban area in Sweden.
4. Next, we compiled the commonly used methodologies for manipulating larval densities in laboratory studies from the literature and found that the majority of laboratory studies identified did not manipulate larval densities below or above the densities observed in nature, suggesting that we have yet to study true life history and physiological responses to low and high population densities during *D. melanogaster* development.
5. This is, to our knowledge, the first direct natural history account of larval density in nature for this model organism. Our study paves the way for a more integrated view of organismal biology which re‐incorporates natural history of model organisms into hypothesis‐driven research in developmental ecology.

## Graphical abstract

Model organisms such as *Drosophila melanogaster* have become powerful laboratory tools, but at the expense of having their natural history overlooked. Here, we provide the first natural history description of larval densities in this species in nature. We also contextualize previous laboratory studies and provide a new powerful framework for future (comparative) studies in insect developmental ecology.

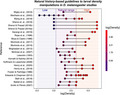

## 1 INTRODUCTION

Model organisms have provided remarkable services to the advancement of fundamental and applied scientific knowledge (Ankeny & Leonelli, 2011). However, the existence of model organisms has become entrenched with the idea of confined and controlled laboratory conditions, and many aspects of model organisms’ natural history have been overlooked (Alfred & Baldwin, 2015). This omission can create challenges for the design and interpretation of studies using model organisms to draw new biological principles that can be applied and tested within and across taxa. *Drosophila melanogaster* Meigen (1830) (Diptera: Drosophilidae) is particularly vulnerable to this caveat because even though this species has become one of the most important scientific model organisms of our time, key natural history information of this species remains to be discovered (Markow, 2015). This is especially true for ecological factors that influence *D. melanogaster* larval development (part of the field of “developmental ecology”).

Broadly, developmental ecology aims to understand how ecological factors experienced early in life affect life history trait expression in current and future generations, as well as population dynamics and species evolution (Watson et al., 2002; West et al., 2005). In insects, one of the biggest challenges in the field of developmental ecology lies on the relativistic nature of methodologies involved in varying population densities early in life (i.e., larval densities) [see (Than et al., 2020)]. This challenge emerges from (a) the various modes observed during (holometabolous) insect development which vary from solitary to gregariousness and in some species, both depending on the developmental stage (e.g., caterpillars) [see for example (Cisternas et al., 2020; Clark & Faeth, 1997; Jin et al., 2016; Klok & Chown, 1999; Morimoto et al., 2018, 2019; Rosa, van Nouhuys, & Saastamoinen, 2017; Stamp & Bowers, 1990) and references therein] and (b) a generalized lack of natural history knowledge for insect larval ecology of many species (Alfred & Baldwin, 2015; Travis, 2020). In this context, what is considered “high” population density in one species will not necessarily represent the same ecological condition in another species, and in fact, there is little consensus on how high (or low) “high (low) density” actually is across species (Than et al., 2020). As a result, it is difficult to standardize methodologies when manipulating larval density, which limits our ability to conduct comparative studies and obfuscate insights into macroevolutionary responses to varying population densities early in life.

One way to overcome this challenge is by gaining insights into the range of larval densities observed for a species in nature, then using these densities as guidelines to establish the ecological meaning of low and high larval densities in laboratory and field studies (Than et al., 2020). This guarantees that, despite quantitative changes used to create high and low densities (e.g., 10 individuals per gram of food in species A versus 100 individuals per gram in species B), the qualitative ecological significance of different manipulations are still comparable (i.e., both species A and B would be in a condition of high density relative to natural conditions). However, even for model organisms (and perhaps worse so for model organisms), natural history information on larval densities is lacking. This is likely in part due to the difficulty in publishing natural history studies but also because we (model organism scientists) often overlook the natural history context from which our isolines and stock colonies originally evolved and continue to evolve (Travis, 2020).

In this study, we collected rotting apples early in the season that were breeding substrates for *D. melanogaster* populations in Sweden, and estimated the natural range of larval densities observed in this model organism. Given that *D. melanogaster* larvae is assumed to be reasonably sessile (Markow, 2015), we measured larval densities at individual fruits. This approach allowed to investigate larval densities in a natural substrate as opposed to the commonly used artificial diets in the laboratory, which are largely composed of mixtures of yeast, cornmeal, and other carbohydrate sources (e.g., molasses) that are absent in natural substrates (see e.g., Matavelli et al., 2015; Rodrigues et al., 2015; Silva‐Soares et al., 2017). Once we had the data of the minimum and maximum larval densities observed in our samples, we collated information from the literature on commonly used manipulation of larval densities in *D. melanogaster* laboratory studies. This allowed us to provide, for the first time, a standardized guideline for larval density manipulation in future studies of developmental ecology in this model organism. This guideline overcomes the relativistic nature of larval density treatments by using natural history information to define what is low and high larval densities, which we propose to be larval densities below and above the minimum and maximum larval densities observed in nature, respectively. Overall, our study has major implications to future eco‐evolutionary experiments using *D. melanogaster* and other model organisms, and paves the way for a more integrated view of organismal biology which re‐incorporates natural history of model organisms into hypothesis‐driven research in developmental ecology (Alfred & Baldwin, 2015; Markow, 2015; Travis, 2020).

## 2 MATERIAL AND METHODS

### 2.1 Sample and data collection

**Figure 1:**
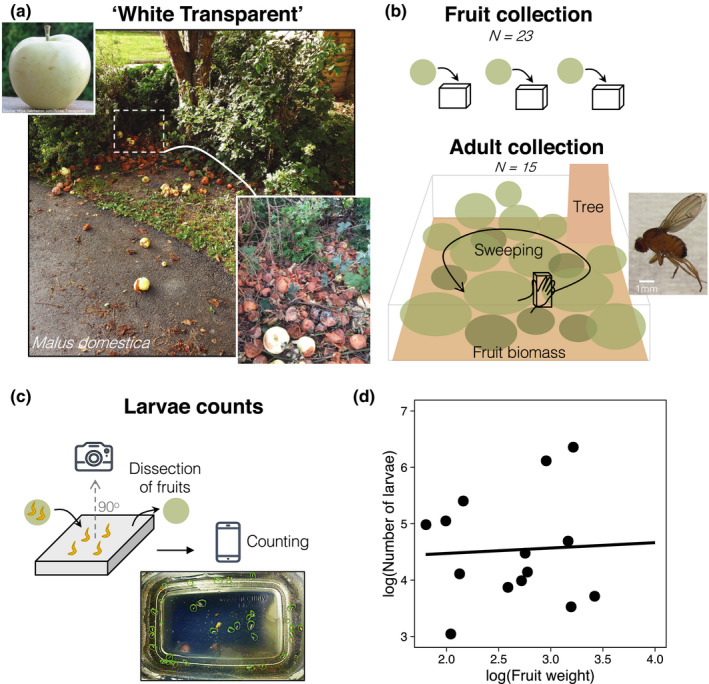
Collection site, methodology, and the relationship between fruit weight and total number of larvae in fruits. (a) Photograph of one of the collection sites in a suburban area of Linköping, Sweden. We randomly collected rotting apples*Malus domestica*var “White Transparent” laying under and nearby the apple tree which were used as breeding substrate for*Drosophila melanogaster*populations. (b) We firstly collected fruit and stored them individually in plastic containers. Next, we sampled the adult population by sweeping a plastic container above the fruit biomass (seeMethodsfor details). (c) We then dissected fruits to collect and count larvae 4 and 7 days after fruit collection. Larvae were placed in a plastic tray with water, and a picture was taken directly above the tray. The picture was then used to count the number of larvae in individual fruits. (d) The log‐transformed relationship between fruit dry weight and the total number of larvae in a given fruit

We randomly collected 23 rotting apples (*Malus domestica,* var “White Transparent”) from four scattered trees around Ryd, a suburban area of the city of Linköping, Sweden (center coordinate of collection site: 58°24ʹ29.6ʺN 15°34ʹ08.7ʺE) in August 2020 (Figure 1a). This variety of apples was ideal for the experiments because of the availability of scattered trees in the area, the distance between trees (>50 m) and also because this is an early‐season cultivar, with fruits available much earlier in the year when temperatures are relatively higher compared with other apple varieties. Fruits were individually stored in plastic trays (c. 12 cm × 6 cm × 5 cm) in outdoor conditions (Figure 1b). We also collected adults from the collection sites (*N *= 15) for species identification, which were frozen at −20°C until further processing. Adult collection was done with a plastic tray (25 cm × 10 cm × 5 cm) that was swept right above (~5 cm) the biomass of rotting fruits; the sweeping was performed three times over a period of 6 min. We dissected the fruits to collect larvae 4 and 7 days after sample collection. This approach allowed us to count larvae that might not have been visible in day 4. After larval picking with tweezers and a wooden pick, we added 5 ml of tap water into the plastic tray to keep larvae in the bottom of the tray (and not on the sides). We placed the tray onto a black board and took a picture of the collected larvae in the plastic tray using a Samsung Note 9 Rear Camera directly above the tray, which allowed us to use inbuilt Samsung Note software to manually count the number of larvae (see Figure 1c). After the last larval collection time point, fruits (*N = *21) were frozen −20°C for 48 hr and dried for 8 hr at 150°C in a commercial oven in small metal (muffin) trays, after which dried fruits were placed in sealable plastic bags and weighted in a Denver Instrument^®^ scale with 0.01 g precision. We collected morphometric values of adult wet weights using a Sartorius scale CP225D with 0.0001 g precision which allowed us to measure variance in adult (male) body size, a common proxy of the potential variance in male mating success and opportunity of sexual selection in *Drosophila* (e.g., Lefranc & Bundgaard, 2000; Morimoto et al., 2016). Images were taken to confirm adult taxonomy in an Olympus SZX16 stereoscope coupled with a wireless Moticam camera with inbuilt software. Adult taxonomic identity was given with morphological characteristics of the specimens, and following identification resources from the literature (e.g., Yuzuki & Tidon, 2020). Direct identification of fruit fly larvae at the species level can be cumbersome without (or even with) molecular tools, and we cannot completely rule out that some of the larvae belongs to other fly species. However, we only found *D. melanogaster* adults in our sweeping samples (apart from two parasitoid wasps individuals), giving us confidence that the observed larvae belong to this species. Our sweeping collected 15 males and one female; we focused on males excluded the female observation since no summary or statistics is possible with *N* = 1 (we discuss potential reasons for this in the Discussion section). We estimated the total number of larvae per fruit as the sum of the number of larvae in each fruit after the two time points of collection. Larvae *per* gram of fruit was calculated by dividing the total number of larvae in each fruit by the fruit weight.

### 2.2 Literature search and statistical analysis

#### 2.2.1 Literature search

We searched the *Drosophila* literature for recent studies that manipulated larval density in laboratory contexts. We firstly used a pragmatic approach whereby we performed a Web of Science search for papers (query: Topic: Larval density AND *Drosophila melanogaster*) which returned 205 papers, 50 of which were published in the last 5 years. From these, 15 directly manipulating larval density in *D. melanogaster*. We also manually added 20 references from studies prior to 2015 (in fact, dating back to 1955) to enrich the scope of our analysis. In total, we had 35 papers, which although is unlikely to account for every paper ever published on *D. melanogaster* larval density, it certainly covers the range of most common manipulations of larval density in laboratory experiments. We read through the Methods section of all 35 papers to extract the data on the manipulations of larval density (e.g., eggs/mL, eggs/g of diet). When the information provided was eggs or larvae *per* vial and no information on the quantity of food *per* vial was given, we still recorded the study but could not accurately calculate estimates of larval density. Our literature survey is summarized in Table 1.

**Table 1:** Summary table of the 35 identified studies manipulating *Drosophila melanogaster* larval density in the laboratory

Treatment	Eggs/larvae (total)	Density (larva or eggs/g or ml)	References
Low, Medium, and High Densities	15, 75, 200 larvae	2, 9, 25 larvae/ml	Amitin and Pitnick (2007)
Continuous (low to high)^b^	NA	NA	Atkinson (1979)
Low, Medium, and High Densities	10–20, 50–70, 150–170	2.5, 10, 26.7 larvae/ml	Baldal et al. (2005)
Low and High Densities	225, 420 larvae	5, 70 larvae/ml	Bath et al. (2018)
Crowded and Uncrowded^a^	NA	NA	Bierbaum et al. (1989)
Low and High Densities^a^	NA	NA	Bubli et al. (1998)
Semi‐continuous	50, 100, 200, 300, 400, 600, 800, 1,000 larvae	6.25, 12.5, 25, 37.5, 50, 75, 100, 125 larvae/ml	Edward and Chapman (2012)
Low and High Densities^a^	35, 200 eggs	NA	Graves and Mueller (1993)
Low, Medium, and High Densities	NA	5, 60, 300 eggs/ml	Henry et al. (2018)
Low, Medium, and High Densities	NA	5, 60, 300 eggs/ml	Henry et al., (2020)
Low, Medium, and High Densities	100–200, 250–500, 1,200–1,500	3, 9, 27 offspring/ml	Hoffmann and Loeschcke (2006)
Low and High Densities	40, 100, 350 eggs	5, 20, 70 eggs/ml	Horváth and Kalinka (2016)
Low and High Densities	60–80, <1,000 larvae	NA	Joshi et al. (1998)
Crowded and Uncrowded	150, 900 eggs	NA	Klepsatel et al. (2018)
Low and High Densities^a^	NA	NA	Leips and Mackay (2000)
Semi‐continuous	10, 20, 40 larvae	2.5, 5, 10 larvae/ml	Lewontin (1955)
Low and High Densities	300–400, 3,000 eggs	NA	Lushchak et al. (2019)
Low, Medium, and High Densities	20, 90, 210 larvae	2, 9, 21 larvae/ml	McGraw et al. (2007)
Low and High Densities	10, 100 eggs	1.5–15 eggs/ml	Moghadam et al. (2015)
Low and High Densities	200, 400 larvae	4, 100 larvae/ml	Morimoto et al. (2016)
Low and High Densities	200, 400 larvae	4, 100 larvae/ml	Morimoto et al. (2017)
Continuous (low to high)^a^	4–125 larvae	7–170 larvae/ml	Moya and Castro (1986)
Low and High Densities	50, 150 larvae	7.1, 21.5 larvae/ml	Roper et al. (1996)
Low and High Densities	60–80, 1,000–1,500 eggs	NA	Santos et al. (1997)
Crowded and Uncrowded	70, 600, 1,200–1,500 eggs	12, 270, 400 eggs/ml	Sarangi et al. (2016)
Crowded and Uncrowded	60–80, 800	1, 535 eggs/ml	Shenoi and Prasad (2016)
Crowded and Uncrowded	60–80, 800	1, 535 eggs/ml	Shenoi, Ali et al. (2016)
Crowded and Uncrowded	60–80, 800	1, 535 eggs/ml	Shenoi, Banerjee, et al. (2016)
Crowded and Uncrowded^a^	NA	NA	Sokolowski et al. (1997)
Low and High Densities^a^	NA	NA	Sørensen and Loeschcke (2001)
Semi‐continuous	NA	1, 6, 20, 40, 60 larvae/cm^3^	Wang et al. (2018)
Semi‐continuous	1, 2, 4, 8, 16, 32 larvae	0.12, 0.25, 0.5, 1, 2, 4 larvae/g	Wertheim et al. (2002)
Low and High Densities	200, 400	4, 100–200 larvae/ml	Wigby et al. (2015)
Semi‐continuous	45–242 larvae	6.5–34.5 larvae/ml	Fowler and Partridge (1986)
Low and High Densities	60–100 eggs	NA	Mueller et al. (2004)

Abbreviation: NA, not available.

^a^ Indirect manipulation of larval density via manipulations of adult density for oviposition.

^b^ Indirect quantification of larval density through adult emergence data.

#### 2.2.2 Statistical analysis

We used two‐tailed binomial tests to assess whether the number of fruits infested with larvae deviated from the expected probability of 0.5. We chose 0.5 expected probability for two reasons: (a) it allowed us to indirectly infer the level of infestation in our sample. If infestation level was high, then probability of findings fruits that were infected in our sample should be higher than 0.5; (b) it allowed us to confidently compare weights of fruits with and without larvae. This is because a probability of 0.5 suggested that we did not have an overrepresentation of fruits with larvae, which could introduce biases in our comparisons of fruit weight and fruit infestation (i.e., with and without larvae). We used one‐way ANOVA to test for differences in average dry weight of fruits that were infested versus noninfested. For those fruits that were infested, we used general linear model to investigate the relationship between total number of larvae (dependent variable) onto fruit dry weight (independent variable), given that one could hypothesize that larger fruits would likely have higher larval densities. We log‐transformed both variables to improve fit of the model verified using diagnostic plots. Summary statistics was provided for all samples (i.e., infested and noninfested fruit weights, larval total number and per gram of fruit, as well as adult weights) (see Table 2). All data were analyzed in R software version 3.6.2 (R Development Team, 2010) while plots were made using the “ggplot2” package (Wickham, 2016).

**Table 2:** Summary of the data collected in this study

Variable	Min	Max	Mean	Median	*SE*
Infested fruit	6.06	30.6	15.8	15.4	2.09
Noninfested fruit	6.52	53.2	20.7	16.3	6.17
Total larvae	21	577	148	75.5	44.6
Larvae per gram	1.34	25.6	10.8	5.1	2.66
Male size	0.61	2.7	1.69	1.715	0.142

## 3 RESULTS

### 3.1 Variation in *Drosophila melanogaster* larval density in natural conditions

The frequency of infested fruits did not deviate from the probability of 0.5 (Binomial test: successes = 15, trials = 23, *p*‐value = .21), suggesting that infestation level was not particularly high. There was no difference in average dried weight between infested and noninfested fruits (*Infested* vs. *noninfested:* *F*_1,19_ = 0.875, *p* = .361, Table 2 and Table S1) suggesting that flies do not necessarily prefer larger fruits—which could potentially represent a better substrate—upon which to lay eggs. In infested fruits, the (log) relationship between fruit weight and the number of larvae was not statistically significant and was close to 0 (*Slope estimate* = 0.094, *standard error of slope* = 0.518, *t‐value* = 0.183, *p‐value* = .857; Table S2, Figure 1d), suggesting that flies do not necessarily lay more eggs in larger fruits. Importantly, larval density varied between 1.34 larvae/g and 25 larvae/g (Table 2), revealing that the range of natural larval densities can vary greatly. Likewise, male adult weight varied between 0.61–2.7 mg, suggesting ample body size variation in the population. Since body size is a proxy for male mating success, this finding suggests that there likely exists strong opportunity for sexual selection in the population.

### 3.2 How high is high density in *Drosophila* studies of larval development?

**Figure 2:**
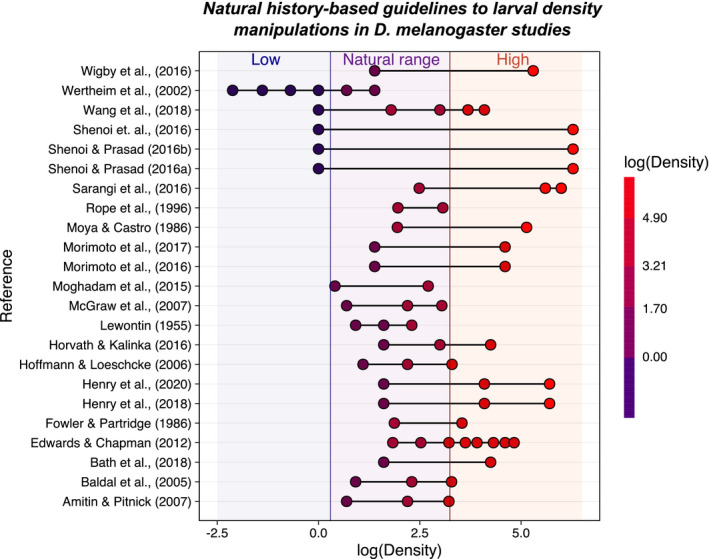
A natural history‐based guideline to larval density manipulations in*Drosophila melanogaster*studies. We collated the information from the literature on laboratory studies manipulating*D. melanogaster*larval density. We then plotted the (log) densities used in these studies while mapping the natural range of densities observed in our natural history observations for this species (purple region). Based on this, we classified as “Low density” the larval densities that were below that minimum larval density observed in our study (blue region). Likewise, we classified as “High density” the larval densities that were above the maximum larval density observed in our study (red region). Each point represents an identified density used in the study [although in some cases, in‐between values could have been possible but were not directly measured; as in, e.g., the high density treatment in (Wigby et al., 2015)]

We compiled a list of 35 studies that manipulated larval density in *D. melanogaster* (see Table 1 and Methods for details). From these, information on larval (or egg) density was available from 23 (~66%), which allowed us to compare how the larval density manipulation in laboratory experiments related to the natural range of larval densities observed in this study. Strikingly, we found that the majority these studies (22 out of 23) failed to either create densities that were lower or higher than the minimum and maximum densities observed in nature, respectively, or only used densities within the observed natural range without exploring lower and/or higher densities (Figure 2). For example, while Morimoto et al., 2016 and 2017 claimed to have studied low and high larval densities, our results suggest that their larval density manipulations corresponded to larval densities within and above (i.e., high) the natural range, but not below (i.e., low density) (Figure 2). Together, these findings highlight the importance of integrating natural history information into laboratory (and potentially field) experiments in insect developmental ecology.

## 4 DISCUSSION

We provide the first direct natural history observation of *D. melanogaster* larval density in nature. Our findings provide an ecological context very much needed for *D. melanogaster*, which is a key model system for laboratory experiments but with surprisingly few observations of its natural history. We also contextualized these data into the broader significance to the field of developmental ecology by combining the data with a review of the common larval density manipulations used in population density studies using *D. melanogaster* in the laboratory. We showed that the majority of studies identified here failed to manipulate larval densities to ranges below and/or above the densities observed in nature and therefore may not necessarily identify ecological responses to extreme larval densities beyond those densities observed in natural populations. This is, to our knowledge, the first detailed description of *D. melanogaster* larval density in nature and has important implications to future studies (in the laboratory or in the field) on developmental ecology of insects. We hope that our approach will inspire future studies to characterize larval densities of other species, which in turn will allow for proper comparative studies in developmental ecology.

The challenge in the field of insect life history responses to population densities early in life lies on the relativistic nature of the definition of population density (see Introduction). Here, we integrated the manipulations of larval density from previous studies in *D. melanogaster* larval density with our natural history findings for the upper and lower observed larval densities for this species in nature. This is, to our knowledge, the first natural history‐driven characterization of low and high densities in this model organism (note that (Atkinson, 1979; Atkinson & Shorrocks, 1984) inferred larval densities indirectly from adult emergence data).

What does this mean to the field of insect developmental ecology? The relativistic nature of the terminology used in insect density‐dependent studies precludes comparative analysis, hampering our ability to understand species‐specific adaptations and other evolutionary responses to population densities in macroevolutionary scales. Here, we provided the first step toward an approach which can resolve this challenge. By incorporating natural history data into our experimental designs, we can truly assess species responses to population densities which vary within, below, and above the population densities observed in nature. In doing so, we will be able to compare responses between species under similar qualitative ecological conditions (e.g., high population density), even if quantitative differences exist to generate these ecological conditions (i.e., differences in the number of individuals needed to generate high densities between species).

Does this mean that past studies that in hindsight failed to manipulate larval densities below or above the natural range of the species have less “worth”? Absolutely not. For once, previous studies were the motivation underlying the need for a proper methodology which incorporates natural history. Moreover, previous studies collectively cover *D. melanogaster* responses to a wide range of densities that can serve as a compass for future studies. For instance, we now know that high larval densities are likely to pose a significant chemical challenge to individuals due to the accumulation of toxic waste (e.g., ammonia) (e.g., Belloni et al., 2018) and a trade‐off between larval developmental rate and feeding efficiency (see Joshi et al., 2001 and references therein). We anticipate that even studies using the natural history‐based guidelines proposed here should still display responses in similar directions to those observed in past studies, even if the magnitude of the effects vary. The value in this definition though, lies on allowing for comparative studies that provide macroevolutionary insights while maintaining ecological significance during the interpretation of the results.

Our sampling method resulted in the collection of mostly males (see “Materials and Methods”). We believe this is because *D. melanogaster* males stay on the surface of the fruits and are territorial, guarding particular patches waiting for females to arrive (see Videos S1 and S2; Hoffmann & Cacoyianni, 1989). It is also possible that, by guarding a territory, males can mate with newly emerged females with potential mating benefits (but see also (Hoffmann & Cacoyianni, 1990) for discussion on mating success benefits of male territoriality). Females, on the other hand, were observed laying eggs inside the fruits (through the cracks and opening caused during the fall from the tree as well as decaying). This degree of spatial separation likely made males more likely to be trapped by our sweeping. This has no effect on the interpretations of our findings.

As with any scientific quest, our study has limitations, which are limitations that are also shared with laboratory and field experiments on insect developmental ecology. For instance, we characterized *D. melanogaster* larval densities in a natural setup, with data collected from rotting apples that were present in a suburban area in Sweden. However, larval density likely varies according to the nutritional value of the substrate. In richer substrates with higher carrying capacity (e.g., higher protein content), larvae develop faster and individuals grow to a bigger body size, suggesting that these substrates can in theory support higher larval densities (e.g., Klepsatel et al., 2018; Nguyen et al., 2019; Silva‐Soares et al., 2017). Thus, it is reasonable to expect that similar effects are present in nature, where different fruits have different nutritional profiles and decaying dynamics, making some fruits better substrates than others. This is particularly relevant for polyphagous species (such as *D. melanogaster*) in which the natural range of larval densities is defined based on all substrates that the species can use for development. In addition to the diet, it is also possible that latitudinal gradients and fruit availability also influence the range of larval densities in populations of cosmopolitan species (such as *D*. *melanogaster*). These limitations shall be uncovered with more natural history studies. Nonetheless, our study is the first to directly address an important yet overlooked gap in our natural history knowledge of one of the most important model organisms in science, providing an important natural history‐guidelines for future studies in the field. Moreover, the approach taken in this study is a stepping‐stone toward integrative and comparative studies designed to better understand insect developmental ecology of insects.

## 5 CONCLUSION

Our findings demonstrate that natural history data are crucial for the design and interpretation of (comparable) studies manipulating larval density in insects. The approach used here shall overcome major limitations in the field of insect developmental ecology and generate important insights into how species respond to varying population densities during early life.

## CONFLICT OF INTEREST

The authors have no conflict of interests to declare.

## AUTHOR CONTRIBUTION

**Juliano Morimoto:** Conceptualization (lead); Data curation (lead); Formal analysis (lead); Investigation (lead); Methodology (lead); Project administration (lead); Resources (lead); Visualization (lead); Writing‐original draft (lead); Writing‐review & editing (equal). **Zuzanna Pietras:** Conceptualization (supporting); Investigation (supporting); Methodology (supporting); Visualization (supporting); Writing‐original draft (supporting); Writing‐review & editing (equal).

## Supporting information

Supplementary Material

Video S1

Video S2

## Data Availability

Raw data available in Dryad: https://doi.org/10.5061/dryad.dfn2z34zx
