# Supplementary material for: Natural history of model organisms: The secret (group) life of Drosophila melanogaster larvae and why it matters to developmental ecology
Source: Ecol Evol. 2020 Nov 10;10(24):13593–601. doi: 10.1002/ece3.7003 (PMC7771115; doi:10.1002/ece3.7003)
Supplement: Supplementary file 1 — Supplementary Material [file ECE3-10-13593-s001.docx]

**Supplementary material: “Natural history of model organisms: the secret (group) life of *Drosophila melanogaster* larvae and why it matters to developmental ecology”**

Authors: Juliano Morimoto^1,*^, Zuzanna Pietras^2^

Authors’ Affiliations:

1 School of Biological Sciences, University of Aberdeen, Zoology Building, Tillydrone Ave, Aberdeen AB24 2TZ

2 Department of Physics, Chemistry and Biology (IFM), Linköping University, Sweden

*Correspondence: [juliano.morimoto@abdn.ac.uk](mailto:juliano.morimoto@abdn.ac.uk)

**Table S1. Complete output for the analysis of differences in weight between infested and non-infested fruits.**

| **Factors** | **Df** | **Sum Sq** | **Mean Sq** | **F-value** | **p-value** |
| --- | --- | --- | --- | --- | --- |
| Infested vs non-infested fruits | 1 | 110.32 | 110.32 | 0.8753 | 0.3612 |
| Residuals | 19 | 2394.83 | 126.04 |  |  |

**Table S2. Complete output for the analysis of the relationship between fruit weight and number of larvae (both variables log-transformed).**

| **Coefficients** | **Estimate** | **std error** | **t-value** | **p-value** |
| --- | --- | --- | --- | --- |
| (Intercept) | 4.29911 | 1.39274 | 3.087 | **0.0094** |
| log(fruit weight) | 0.09488 | 0.51862 | 0.183 | 0.8578 |

**Supplementary Media 1 (mp4 video). Video of males interacting in seemingly territoriality contests.** Note wing threatening and chasing in males throughout the screen.

**Supplementary Media 2 (mp4 video). Video of males interacting in seemingly territoriality contests.** Note chasing in the pair of males in the centre of the screen.
